# Tibial tubercle torsion is associated with patellar height when measured by computed tomography

**DOI:** 10.1002/jeo2.70258

**Published:** 2025-05-06

**Authors:** Joseph D. Giusto, Janina Kaarre, Yongji Kim, Jae‐Sung An, Sally LiArno, Faizan Ahmad, Matthieu Ollivier

**Affiliations:** ^1^ Department of Orthopaedic Surgery, UPMC Freddie Fu Sports Medicine Center University of Pittsburgh Medical Center Pittsburgh Pennsylvania USA; ^2^ Department of Orthopaedics, Institute of Clinical Sciences Sahlgrenska Academy University of Gothenburg Gothenburg Sweden; ^3^ Institut du Mouvement et de l'appareil Locomoteur (IML) Hôpital Sainte‐Marguerite, AP‐HM Marseille France; ^4^ Department of Orthopaedics Faculty of Medicine Juntendo University Tokyo Japan; ^5^ Department of Joint Surgery and Sports Medicine Graduate School of Medical and Dental Sciences, Tokyo Medical and Dental University Tokyo Japan; ^6^ Stryker Mahwah New Jersey USA

**Keywords:** CT, Insall‐Salvati index, patellar height, patellar instability, tibial tubercle torsion, TTTG

## Abstract

**Purpose:**

To establish an average tibial tubercle (TT) torsion angle from computed tomography (CT) scans of patients without known patellofemoral instability and investigate whether TT torsion angles would differ based on demographics, tibial tubercle‐trochlear groove (TT‐TG) distance and patellar height.

**Methods:**

The Stryker Orthopaedics Modeling and Analytics (SOMA) database was queried for patients with CT scans and available measures related to patella and TT position. The mean TT torsion angle was compared in patients with an increased and normal TT‐TG distance (≥20 vs. <20 mm) and patellar height (Insall–Salvati [IS] index ≥1.3 vs. <1.3). Measurements of sulcus angle, patellar inclination angle, congruence angle, trochlear groove depth and long limb axis alignment were assessed.

**Results:**

A total of 886 knees from 499 patients within the SOMA database were included, with a mean age of 59.4 ± 16.5 years and 238 (48%) females. The mean TT torsion angle for all patients was 24.7 ± 5.2°. Females had a significantly higher mean IS index (1.24 vs. 1.18), TT‐TG distance (13.8 mm vs. 11.8 mm) and TT torsion angle (25.5° vs. 24.0°) compared to males. The mean TT torsion angle for patients with a TT‐TG distance ≥20 mm and <20 mm was 24.7° in both groups (*p* = n.s.). There was a significantly greater TT torsion angle in patients with an IS index ≥1.3 (26.6°) compared to those with an IS index <1.3 (24.0°) (*p* < 0.001). A weak and positive correlation was found between TT torsion angle and IS index (*r* = 0.242, *p* < 0.001), but not with other measurements.

**Conclusion:**

The mean TT torsion angle for patients without known patellofemoral instability was 24.7° and increased TT torsion angles were associated with increased patellar height. An association between TT torsion and TT‐TG was not found. Findings of the current study describe the relationship between morphologic assessments of the patellofemoral joint using CT.

**Level of Evidence:**

Level IV, cohort study.

**Abbreviations**
3D: three‐dimensional
ANOVA: analysis of variance
BMI: body mass index
CT: computed tomography
IS: Insall–Salvati
MPFL: medial patellofemoral ligament
MRI: magnetic resonance imaging
SOMA: Stryker Orthopaedics Modeling and Analytics
TT: tibial tubercle
TTO: tibial tubercle osteotomies
TT‐TG: tibial tubercle‐trochlear groove

## INTRODUCTION

Several factors are associated with an increased risk of patellofemoral instability, including younger age, trochlear dysplasia, increased tibial tubercle (TT)‐trochlear groove (TT‐TG) distance, patella alta and lateral patellar displacement [7, 8, 10, 13]. In patients with recurrent patellar dislocation, surgery may be indicated to correct anatomic risk factors. Medial patellofemoral ligament (MPFL) reconstruction, tibial tubercle osteotomies (TTO) and trochleoplasties are common options in such cases [2, 16, 17, 19]. However, obtaining accurate measurements that influence surgical planning, such as the TT‐TG distance, can be challenging when trochlear dysplasia is present [20, 23].

TT position has subsequently been examined using other measures like TT rotation [11, 18], but this may be confounded by TT lateralization. As a result of these limitations, a TT torsion angle, which is measured between the transverse axis of the TT and the tangential axis of either the posterior femur or tibia on an axial view [4, 14], has been proposed to better characterize TT external rotation. Higher TT torsion angles using magnetic resonance imaging (MRI) have been found in groups with patellofemoral instability compared to unaffected controls [4, 14]. A biomechanical cadaveric study has also found improved patellar stability when reducing TT torsion [5]. However, a reference TT torsion angle using computed tomography (CT) scans in an unaffected population is not well established despite its use in MRI. Establishing the reference CT value for another anatomic feature associated with patellofemoral instability could be beneficial for the clinical evaluation of patients who may have less reliable measurements of other risk factors (i.e. less reliable TT‐TG due to trochlear dysplasia). Furthermore, associations between TT torsion angle and other measures of patellofemoral instability are not well described using CT.

The purpose of this study was to use CT scans to determine the average TT torsion angle in patients without known patellofemoral instability and investigate whether TT torsion angle would differ between patients of varying demographics, TT‐TG distances, and patellar height. Our hypothesis was that few differences in radiographic measurements would exist between varying demographics, but TT torsion would be increased in patients with an increased TT‐TG distance and patella alta given its previously reported association with patellofemoral instability [4, 14].

## PATIENTS AND METHODS

### Patient population

The Stryker Orthopaedics Modeling and Analytics (SOMA) system (Stryker®) was queried for all patients with available CT scans and measurements related to patellar height and TT position. The SOMA database was developed using a global sample of over 3600 patients who received CT scans and three‐dimensional (3D) bone models for medical indications such as CT angiography (70%), polytrauma (20%) and other reasons, including total knee replacements (10%) [21]. Patients without measurements of TT torsion, TT‐TG distance and patellar height as measured by the IS index were excluded. Additionally, CT scans with greater than 10° of knee flexion as measured by the tibio‐femoral angle in the sagittal plane were excluded. Skeletal maturity was not assessed for study eligibility and a minimum age was not implemented for study inclusion. Demographic variables, including age, sex, height, weight, body mass index (BMI) and ethnicity, were obtained from the database along with the radiographic measurements.

### Radiographic measurements

**Figure 1:**
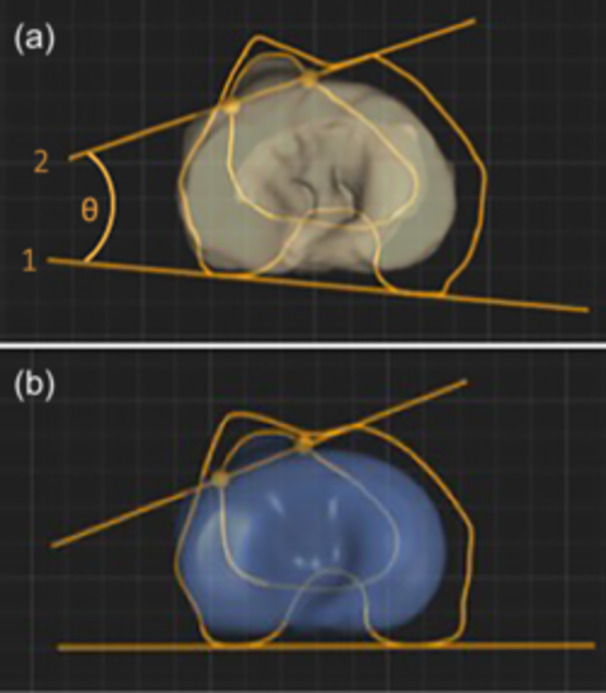
Calculation of the tibial tubercle (TT) torsion angle. (a) A patient image is used to manually calculate TT torsion using an axial computed tomography (CT) slice. (1) The cut in which the posterior femoral condyles are best visualized is used to create the posterior femoral condylar axis. (2) To determine the TT axis, a line is drawn through the intersection of two points marking the medial and lateral borders of the tibial tubercle. (θ) The TT torsion angle is then calculated on the medial side of the two axes. (b) Example of TT torsion calculation using three‐dimensional bone modelling of CT scans from SOMA automated software (24.2°).

The TT torsion angle was calculated as the angle between the TT transverse axis, which was defined by the medial and lateral borders of the TT in an axial slice, and a second axis tangential to the posterior femoral condyles on an axial CT slice (Figure 1) [4]. The TT‐TG distance and IS index were also obtained [8, 25]. The TT‐TG distance was calculated using axial slices as the distance between the TT and the deepest point on the trochlear groove. The IS index was calculated in a sagittal slice by dividing the length of the patellar tendon (distance from the inferior patellar pole to the TT when using CT) by the length of the patella. Additionally, other patellofemoral measurements (sulcus angle, patellar inclination angle, congruence angle and trochlear groove depth) and long limb axis alignment measurements (lateral distal femoral angle, medial proximal tibial angle, joint line convergence angle and hip–knee–ankle angle) were included for patients with available data.

The mean TT torsion angle was grouped by literature references of increased TT‐TG (≥20 mm) and IS index (≥1.3), which is common in patients with patellofemoral instability [8, 9, 13, 25]. A CT cutoff value for an increased TT‐TG distance of 20 mm has been well described [8]. An IS index cutoff value of 1.3 was used given previous reports of cutoffs ranging from 1.2 to 1.5 when using MRI and a pooled mean IS index among 10 studies of 1.31 for patients with patellofemoral instability [9, 25]. The IS index was chosen as the measure of patellar height given reports that it is more reliable than the Caton‐Deschamps index and Blackburne‐Peel ratio with moderate and higher inter‐method reliability between CT and MRI (intra‐class correlation coefficient = 0.70) [24]. Additionally, the IS index is not influenced by the proximal tibial plateau and may be better related to TT position. Cutoff values were also estimated for sulcus angle (153°), patellar inclination angle (17°), congruence angle (26°), and trochlear groove depth (3.6 mm) based on a previous study investigating the CT threshold values for these measurements in patients with and without patellofemoral instability [6].

### Measurement assessment

Fifty images (25 left and 25 right knees, respectively) were obtained from the database, and the drawn TT torsion angles were re‐calculated to assess their comparability with the angles obtained from the SOMA database, which were calculated using an automated software (Supporting Information S1: Table 1). Outliers for included measurements were additionally calculated using the interquartile ranges and are listed in Supporting Information S1: Table 2.

### Statistical analyses

Categorical variables are presented as counts (n) and proportions (%), while ordinal and continuous data are presented as means and standard deviations (SDs). A Gaussian distribution was used to define the 95% central values of TT torsion. A Shapiro–Wilk test was performed to determine the normality of the data. Independent samples *t*‐tests were used for parametric variables and Mann–Whitney *U* tests for non‐parametric variables when comparing differences between groups. A one‐way analysis of variance (ANOVA) or Kruskal–Wallis test was performed to compare differences when more than two groups were present. Correlations between TT torsion angle and other patellofemoral measurements were assessed using a bivariate (Pearson's) correlation analysis. A linear regression analysis was performed to assess the relationship between age and TT torsion angle, TT‐TG distance and IS index. Statistical significance was determined using a *p* value cutoff of <0.05 and analyses were performed using SPSS version 29.0.1.0 (IBM).

## RESULTS

**Figure 2:**
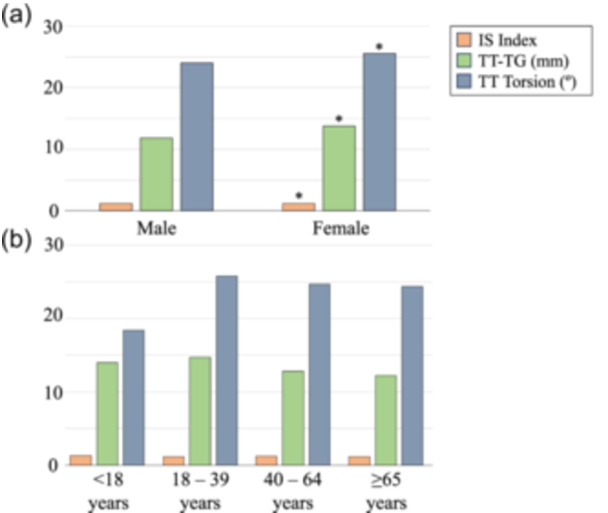
Demographic comparisons of radiographic measurements. (a) Sex: male (*n* = 463), female (*n* = 423). (b) Age in years: <18 (*n* = 8), 18–39 (*n* = 103), 40–64 (*n* = 371) and ≥65 (*n* = 367). *Significantly higher value detected compared to males (*p* < 0.05). CT, computed tomography; IS, Insall‐Salvati; TT, tibial tubercle; TT‐TG, tibial tubercle‐trochlear groove. Measurements described for all included CT scans (not individual patients).

After screening the SOMA database, a total of 886 knees from 499 patients (48% female) met the inclusion criteria with a mean TT torsion angle of 24.7 ± 5.2° (95% central values 14.4–35.1°) (Table 1). The mean IS index was 1.21 ± 0.26, and the mean TT‐TG distance was 12.8 ± 9.3 mm. The average IS index, TT‐TG and TT torsion were comparable between left and right CT scans (*p* = n.s.). A significantly higher IS index (1.24 vs. 1.18), TT‐TG distance (13.8 mm vs. 11.8 mm) and TT torsion angle (25.5° vs. 24.0°) were found in females compared to males (*p* < 0.001, *p* = 0.006, *p* < 0.001, respectively; Figure 2a). TT torsion angles were significantly different between age groups (<18 years: 18.4°; 18–39 years: 25.8°; 40–64 years: 24.7°; ≥65 years: 24.4°, *p* < 0.001) along with TT‐TG (<18 years: 14.0 mm; 18–39 years: 14.7 mm; 40–64 years: 12.8 mm; ≥65 years: 12.2 mm, *p* = 0.010) (Figure 2b). A linear regression analysis revealed no significant association between age and TT torsion angle (*R* ^2^ = 0.004, *p* = n.s.) nor IS index (*R* ^2^ = 0.001, *p* = n.s.), but a significant relationship between age and TT‐TG (*R* ^2^ = 0.008, *p* = 0.008).

**Table 1:** Baseline characteristics of included patients.

Demographics	*n* = 499 patients
Age (years) (*n* = 480)	59.4 ± 16.5 (11–92)
Sex (male), *n* (%)	261 (52.3)
Weight (kg) (*n* = 332)	69.0 ± 15.5 (33–110)
Height (cm) (*n* = 333)	165.8 ± 9.4 (137–189)
BMI (kg/m^2^) (*n* = 332)	24.9 ± 4.5 (13.8–41.6)
Ethnicity, *n* (%)	
African	5 (1.0)
Asian	226 (45.3)
Caucasian	259 (51.9)
Middle Eastern	9 (1.8)

Radiographic measurements	*n* = 886 measurements
Laterality (right), *n* (%)	455 (51.4)
Patellofemoral measurements	
Sulcus angle (°) (*n* = 811)	155.7 ± 8.2 (128.1–179.3)
Patellar inclination angle (°) (*n* = 298)	8.7 ± 4.9 (0.0–23.1)
Congruence angle (°) (*n* = 298)	9.4 ± 10.8 (0.1–161.5)
Trochlear groove depth (mm) (*n* = 811)	3.4 ± 1.3 (0.1–7.5)
IS Index	1.21 ± 0.26 (0.60–3.59)
TT‐TG distance (mm)	12.8 ± 9.3 (0.0–67.0)
TT torsion angle (°)	24.7 ± 5.2 (9.2–39.2)
Long limb axis measurements	
Lateral distal femoral angle (°) (*n* = 797)	86.3 ± 1.9 (80.3–93.5)
Medial proximal tibial angle (°) (*n* = 422)	85.0 ± 2.2 (77.5–90.9)
Joint line convergence angle (°) (*n* = 422)	1.1 ± 0.9 (0.0–6.1)
Hip–knee–ankle angle (°) (*n* = 428)	178.8 ± 2.4 (168.7–184.7)

*Note*: Data presented as mean ± standard deviation (minimum–maximum) unless otherwise specified. Demographic variables are listed for individual patients as some patients received bilateral imaging.

Abbreviations: BMI, body mass index; IS, Insall–Salvati; kg, kilograms; m, meters; mm, millimeters; n, number of patients; TT, tibial tubercle; TT‐TG, tibial tubercle‐trochlear groove.

No statistically significant difference was found in mean TT torsion angle between knees with a TT‐TG distance ≥20 and <20 mm (24.7° vs. 24.7°, *p* = n.s.; Table 2). However, a significantly greater TT torsion angle was found in patients with an IS index ≥1.3 compared to those with an IS index <1.3 (26.6° vs. 24.0°, *p* < 0.001), with a mean difference of 2.6° between groups. When comparing TT torsion angle between patients above and below the CT threshold values for sulcus angle, patellar inclination angle, congruence angle, and trochlear groove depth, no significant differences were detected (*p* = n.s.) (Supporting Information S1: Table 3).

**Table 2:** Mean TT torsion angle grouped by TT‐TG distance and IS index.

	TT‐TG < 20 mm (*n* = 704)	TT‐TG ≥ 20 mm (*n* = 182)	*p*	IS index < 1.3 (*n* = 656)	IS index ≥ 1.3 (*n* = 230)	*p* value
Tibial tubercle torsion angle (°)	24.7 ± 5.3 (9.2–39.2)	24.7 ± 4.7 (11.5–35.6)	n.s.	24.0 ± 4.9 (9.2–38.0)	26.6 ± 5.4 (12.4–39.2)	**<0.001** *

*Note*: Data presented as mean ± standard deviation (minimum–maximum).

Abbreviations: IS, Insall‐Salvati; n, number of patients; TT, tibial tubercle; TT‐TG, tibial tubercle‐trochlear groove distance.

* Statistically significant difference (*p* value < 0.05).

A significant correlation was not found between TT torsion angle and sulcus angle (*r* = −0.044, *p* = n.s.), patellar inclination angle (*r* = 0.039, *p* = n.s.), congruence angle (*r* = −0.008, *p* = n.s.) or trochlear groove depth (*r* = 0.048, *p* = n.s.). Likewise, a significant correlation was not detected between TT torsion and measures of long limb axis alignment, including lateral distal femoral angle (*r* = −0.039, *p* = n.s.), medial proximal tibial angle (*r* = −0.037, *p* = n.s.), joint line convergence angle (*r* = −0.053, *p* = n.s.) and hip‐knee‐ankle angle (*r* = −0.021, *p* = n.s.). However, there was a significant weak and positive correlation between TT torsion angle and IS index (*r* = 0.242, *p* < 0.001).

## DISCUSSION

The most important finding of the present study is that the mean TT torsion angle using CT in patients without known patellofemoral instability was 24.7° and increased TT torsion angles were associated with increased patellar height. There was no significant difference in the mean TT torsion angle for patients with a TT‐TG ≥ 20 mm. Secondary findings include a statistically significantly higher IS index, TT‐TG and TT torsion for patients of female sex. Results provide surgeons with a reference CT measurement of TT torsion in patients without known patellofemoral instability. This can be used in the clinical evaluation of affected patients, as they may have increased TT torsion angles [4, 14].

The mean TT torsion angle found in this study sample was interestingly greater than previously reported values. Specifically, prior MRI studies of TT torsion angle have reported average angles of 16.0° and 17.9° in patients with patellofemoral instability and 5.8° and 9.1° in control patients [4, 14]. This discrepancy could be related to differences between MRI and CT imaging and measurement error. It is known that measurements of another patellofemoral instability risk factor, TT‐TG distance, vary between CT and MRI, with CT measurements being greater compared to MRI measurements [3, 12, 22]. This measurement difference has been explained by a higher average knee flexion observed in MRI (11.14°) compared to CT (3.68°) scans [12]. Native knee rotation has also been shown to be independently predictive of differences in measured TT‐TG between MRI and CT, which highlights the complexity of comparing measurements between different imaging modalities [1]. Therefore, higher TT torsion angles observed in the present study could be attributed to increased knee extension and external tibial rotation during CT scans or differences in native knee rotation. However, given the absence of a reliability assessment for included measurements, measurement error is another possible explanation for differences from prior studies.

Female patients had a higher IS index, TT‐TG and TT torsion, all of which are risk factors for patellofemoral instability [4, 7, 8, 13, 14]. These findings suggest that females have anatomic differences in TT position compared to males that may place them at a higher risk for patellofemoral instability. However, given conflicting evidence regarding sex‐based differences in the risk for patellar instability, it is unclear whether the statistically significant findings found in this study are also clinically significant [7, 13]. Statistically significant differences were also detected in TT torsion and TT‐TG based on age. However, only TT‐TG remained significant on linear regression analysis and with just 0.8% of the variance in TT‐TG explained by the variance in age.

A statistically significantly higher TT torsion was found in patients with patella alta (IS index ≥1.3) compared to patients with normal patellar height (IS index <1.3), aligning with one part of our hypothesis. TT torsion angle was also weakly correlated with IS index. One explanation for this finding could be a possible ‘lengthening bias’ in the measurement from the distal patellar pole to the TT. Thus, patients with increased torsion of the tubercle may have had a more lateral position of the TT, causing an increase in the measured distance from the inferior patellar pole to the TT and corresponding increase in the calculated IS index without any change in patella position. A second explanation could be that patients with patella alta had a combined deformity including increased TT torsion. No statistically significant difference was observed in TT torsion when grouped by TT‐TG distances ≥20 and <20 mm. However, prior studies have reported conflicting results on the association between TT‐TG and TT torsion [4, 14]. One study found both TT‐TG and TT rotation to be independently predictive of instability, while another study noted the loss of significant association between TT‐TG and instability on multivariate analysis due to the strong correlation between the two measurements [4, 14]. However, the results of the current study suggest that the TT torsion angle may be independent of TT‐TG and explain why some patients have a lateral and tilted patella in the absence of a TT‐TG abnormality. Surgeons may use this information in the clinical setting as a reference value for an anatomic feature associated with patellofemoral instability, particularly when confidence in other measures such as TT‐TG is low. Evaluations of TT torsion may also be beneficial in patients with isolated patella alta given the association described in the current study. Future studies specifically comparing TT torsion angles measured by CT in both affected and unaffected populations are needed.

This study also had several limitations. Firstly, the mean age of this study population (59.4 years) was considerably older than the patient population typically affected by patellofemoral instability [7, 13], which limits the generalizability of study results. Secondly, the use of CT to calculate TT torsion may have led to measurement error considering that the identification of the TT on CT may have been more challenging compared to MRI. The resolution (slice thickness) of CT scans was not standardized, leading to a possibility for measurement error. Measurements of femoral anteversion and tibial torsion were also not included in the present analysis. Measurement error may bias results as a reliability assessment was not performed using intraclass correlation coefficient calculations, although there were few outliers relative to the sample size. Finally, it is possible that some of the included patients had an unknown history of patellofemoral instability, leading to possible selection bias. However, the mean TT‐TG distance of all included patients (12.8 mm) is comparable to the mean CT measurement of TT‐TG in groups without instability analyzed in prior studies (12.6–12.7 mm) [8, 25], suggesting that the included patient population is representative of patients without patellofemoral instability. Furthermore, very few patients in the included study are likely to be affected by patellofemoral instability given the previously reported low incidence of this pathology [15].

## CONCLUSION

The mean TT torsion angle for patients without known patellofemoral instability was 24.7° and increased TT torsion angles were associated with increased patellar height. An association between TT torsion and TT‐TG was not found. Findings of the current study describe the relationship between morphologic assessments of the patellofemoral joint using CT.

## AUTHOR CONTRIBUTIONS

Yongji Kim and Jae‐Sung An analyzed the literature. Sally LiArno and Faizan Ahmad extracted data from SOMA. Janina Kaarre, Joseph D. Giusto and Matthieu Ollivier wrote the final manuscript. All authors approve the final content of the manuscript.

## CONFLICT OF INTEREST STATEMENT

Matthieu Ollivier is a Newclip educational consultant and receives royalties from Stryker. Sally LiArno and Faizan Ahmad are employees of Stryker. The remaining authors declare no conflicts of interest.

## ETHICS STATEMENT

Ethical approval for this study was obtained via a waiver from the AP‐HM ethics committee (No. PADS23‐167_dgr).

## Supporting information

Supporting information.

## Data Availability

The data that support the findings of this study are available on request from the corresponding author. The data are not publicly available due to privacy or ethical restrictions.
