# Supplementary material for: Tibial tubercle torsion is associated with patellar height when measured by computed tomography
Source: J Exp Orthop. 2025 May 6;12(2):e70258. doi: 10.1002/jeo2.70258 (PMC12053445; doi:10.1002/jeo2.70258)
Supplement: Supplementary file 1 — Supporting information. [file JEO2-12-e70258-s001.docx]

**Supplementary Table 1: Comparison of the TT torsion angles.**

| Left knee  (TT angle °) | Original Angle from the Database | Re-calculation of the Angle |
| --- | --- | --- |
|  | 30.8 | 31.0 |
|  | 25.3 | 25.2 |
|  | 35.7 | 35.5 |
|  | 36.8 | 36.4 |
|  | 30.8 | 30.9 |
|  | 24.3 | 24.5 |
|  | 22.1 | 22.1 |
|  | 26.0 | 25.8 |
|  | 27.2 | 27.3 |
|  | 24.5 | 24.6 |
|  | 26.7 | 26.9 |
|  | 28.0 | 27.9 |
|  | 23.3 | 23.3 |
|  | 30.7 | 30.6 |
|  | 29.0 | 29.1 |
|  | 24.0 | 24.1 |
|  | 35.5 | 35.8 |
|  | 26.6 | 26.7 |
|  | 29.2 | 29.3 |
|  | 27.3 | 27.4 |
|  | 33.5 | 33.6 |
|  | 33.2 | 33.4 |
|  | 38.0 | 38.0 |
|  | 27.7 | 27.4 |
|  | 30.0 | 30.1 |
| **Right knee**  **(TT angle °)** | 27.5 | 27.7 |
|  | 34.2 | 34.3 |
|  | 30.3 | 30.0 |
|  | 22.2 | 22.2 |
|  | 29.5 | 29.4 |
|  | 23.5 | 23.6 |
|  | 30.1 | 30.0 |
|  | 24.7 | 24.5 |
|  | 22.9 | 22.8 |
|  | 27.0 | 27.0 |
|  | 31.4 | 31.3 |
|  | 24.8 | 24.8 |
|  | 33.3 | 33.3 |
|  | 33.5 | 33.4 |
|  | 29.2 | 29.9 |
|  | 25.8 | 25.9 |
|  | 27.8 | 28.0 |
|  | 32.0 | 31.9 |
|  | 19.9 | 19.7 |
|  | 28.4 | 28.3 |
|  | 27.6 | 27.7 |
|  | 21.5 | 21.0 |
|  | 27.6 | 27.5 |
|  | 25.6 | 25.6 |
|  | 33.8 | 34.0 |
| TT = tibial tubercle |  |  |

**Supplementary Table 2:** Measurement outliers

| Measurement | Outlier boundaries | Number of data points below lower limit boundary | Number of data points above upper limit boundary |
| --- | --- | --- | --- |
| **IS Index (n=886)** | 0.66 – 1.69 | 1 | 33 |
| **TT-TG distance (mm) (n=886)** | -12.0 – 36.0 | 0 | 15 |
| **TT Torsion Angle (°) (n=886)** | 11.6 – 38.0 | 6 | 2 |
| **Sulcus angle (°) (n=811)** | 134.7 – 176.3 | 4 | 9 |
| **Patellar inclination angle (°) (n=298)** | -5.5 – 23.0 | 0 | 1 |
| **Congruence angle (°) (n=298)** | -11.5 – 28.2 | 0 | 1 |
| **Trochlear groove depth (mm) (n=811)** | 0.2 – 6.6 | 3 | 6 |
| **Lateral distal femoral angle (°) (n=797)** | 81.7 – 90.9 | 1 | 10 |
| **Medial proximal tibial angle (°) (n=422)** | 79.2 – 90.8 | 2 | 1 |
| **Joint line convergence angle (°) (n=422)** | -1.1 – 2.9 | 0 | 21 |
| **Hip-knee-ankle angle (°) (n=428)** | 172.8 – 184.6 | 5 | 3 |

*Outlier boundaries were calculated using the below definition. The number of data points falling outside the respective boundaries (number of outliers) is listed in the last two columns.*

*Lower outlier boundary = first quartile – (1.5 * interquartile range)*

*Upper outlier boundary = third quartile + (1.5 * interquartile range)*

**Supplementary Table 3:** Mean TT torsion angle grouped by other measures of patellofemoral instability

| Measurement | Tibial Tubercle Torsion Angle (°) | P value |
| --- | --- | --- |
| **Sulcus angle (n=811)** |  |  |
| <153° (n=289) | 25.0 ± 5.2 (11.0 – 39.2) | n.s. |
| ≥153° (n=522) | 24.7 ± 5.1 (9.2 – 39.0) |  |
| **Patellar inclination angle (n=298)** |  |  |
| <17° (n=278) | 24.6 ± 4.9 (10.1 – 36.7) | n.s. |
| ≥17° (n=20) | 26.4 ± 5.0 (17.0 – 36.8) |  |
| **Congruence angle (n=298)** |  |  |
| <26° (n=296) | 24.7 ± 4.9 (10.1 – 36.8) | n.s. |
| ≥26° (n=2) | 26.8 ± 1.9 (25.4 – 28.1) |  |
| **TGD (n=811)** |  |  |
| <3.6mm (n=447) | 24.6 ± 5.1 (9.2 – 39.0) | n.s. |
| ≥3.6mm (n=364) | 25.1 ± 5.2 (11.0 – 39.2) |  |

*Data presented as mean ± standard deviation (minimum – maximum).*

**Statistically significant difference (P value <0.05).*

*TGD: trochlear groove depth, n: number of patients.*
